# Rapid Increase of SARS-CoV-2 Variant B.1.1.7 Detected in Sewage Samples from England between October 2020 and January 2021

**DOI:** 10.1128/mSystems.00353-21

**Published:** 2021-06-15

**Authors:** Thomas Wilton, Erika Bujaki, Dimitra Klapsa, Manasi Majumdar, Maria Zambon, Martin Fritzsche, Ryan Mate, Javier Martin

**Affiliations:** a Division of Virology, National Institute for Biological Standards and Control, South Mimms, Potters Bar, Hertfordshire, UK; b Respiratory Virology and Polio Reference Service, Public Health England, London, UK; c Division of Analytical and Biological Sciences, National Institute for Biological Standards and Control, South Mimms, Potters Bar, Hertfordshire, UK; University of California—San Francisco

**Keywords:** SARS-CoV-2, environmental surveillance, variant B.1.1.7, variant of concern, COVID-19, sewage, vaccine, wastewater, next-generation sequencing, direct detection, B.1.1.7, surveillance

## Abstract

SARS-CoV-2 variants with multiple amino acid mutations in the spike protein are emerging in different parts of the world, raising concerns regarding their possible impact on human immune response and vaccine efficacy against the virus. Recently, a variant named lineage B.1.1.7 was detected and shown to be rapidly spreading across the UK since November 2020. As surveillance for these SARS-CoV-2 variants of concern (VOCs) becomes critical, we have investigated the use of environmental surveillance (ES) for the rapid detection and quantification of B.1.1.7 viruses in sewage as a way of monitoring its expansion that is independent on the investigation of identified clinical cases. Next-generation sequencing analysis of amplicons synthesized from sewage concentrates revealed the presence of B.1.1.7 mutations in viral sequences, first identified in a sample collected in London on 10 November 2020 and shown to rapidly increase in frequency to >95% in January 2021, in agreement with clinical data over the same period. We show that ES can provide an early warning of VOCs becoming prevalent in the population and that, as well as B.1.1.7, our method can detect VOCs B.1.351 and P.1, first identified in South Africa and Brazil, respectively, and other viruses carrying critical spike mutation E484K, known to have an effect on virus antigenicity. Although we did not detect such mutation in viral RNAs from sewage, we did detect mutations at amino acids 478, 490, and 494, located close to amino acid 484 in the spike protein structure and known to also have an effect on antigenicity.

**IMPORTANCE** The recent appearance and growth of new SARS-CoV-2 variants represent a major challenge for the control of the COVID-19 pandemic. These variants of concern contain mutations affecting antigenicity, which raises concerns on their possible impact on human immune response to the virus and vaccine efficacy against them. Here, we show how environmental surveillance for SARS-CoV-2 can be used to help us understand virus transmission patterns and provide an early warning of variants becoming prevalent in the population. We describe the detection and quantification of variant B.1.1.7, first identified in southeast England in sewage samples from London (UK) before widespread transmission of this variant was obvious from clinical cases. Variant B.1.1.7 was first detected in a sample from early November 2020, with the frequency of B.1.1.7 mutations detected in sewage rapidly increasing to >95% in January 2021, in agreement with increasing SARS-CoV-2 infections associated with B.1.1.7 viruses.

## INTRODUCTION

**FIG 1:**
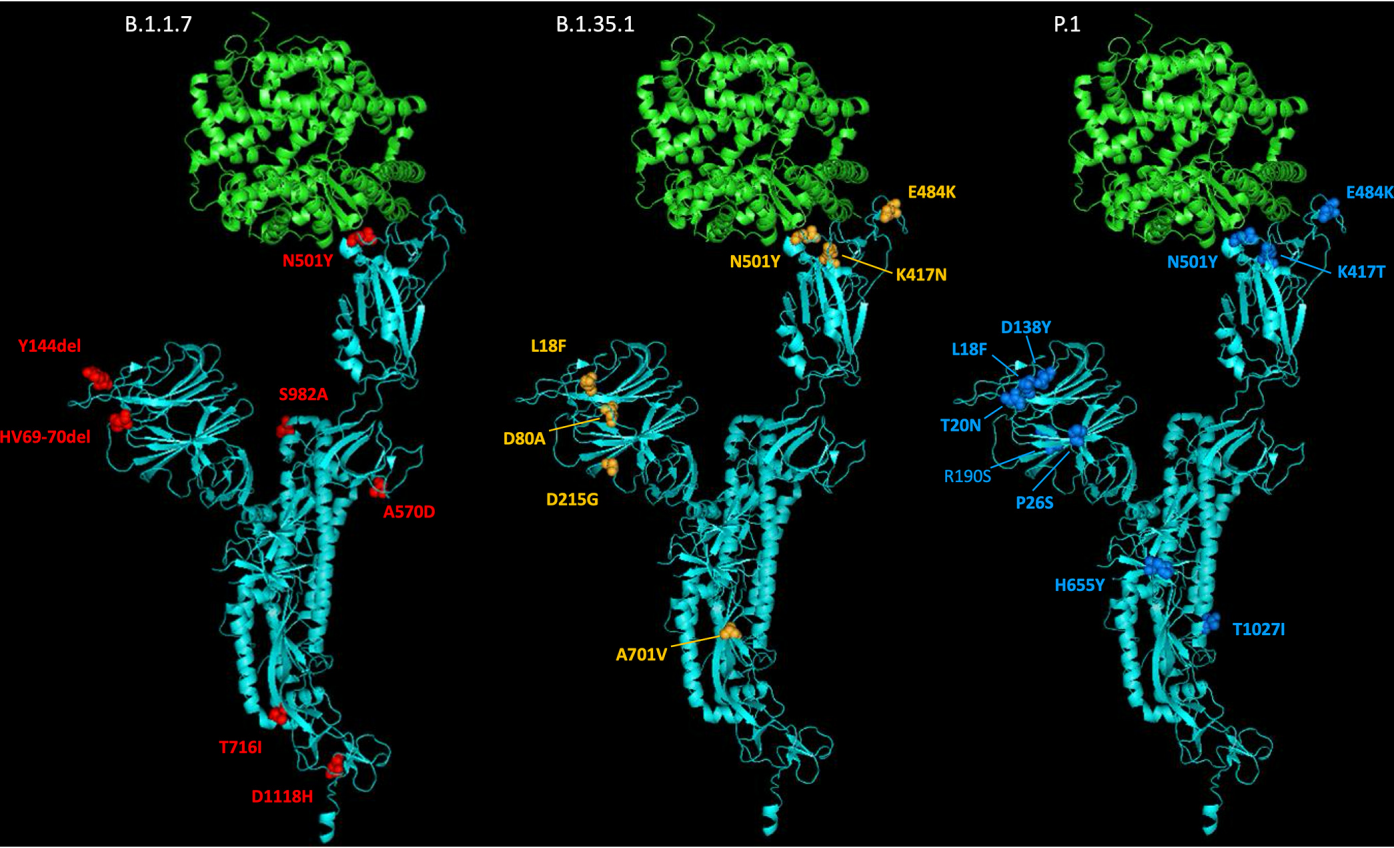
Molecular cartoon diagram of the 3D structure of SARS-CoV-2 spike protein monomer in the open form (cyan) in complex with receptor ACE-2 (green). The image was generated using PyMOL Molecular Graphics System version 1.7.0.3 software (Schrödinger, LLC) using cryo-EM data (Protein Data Bank accession number 7DF4 [36]). Amino acid substitutions found in B.1.1.7 (red), B.1.351 (orange), and P.1 (blue) variants are shown.

A novel SARS-CoV-2 lineage B.1.1.7 was recently identified and shown to be rapidly expanding across the UK, particularly in London and in the southeast and the east of England (1, 2). This lineage was detected in November 2020 and likely originated in September 2020 in Kent, southeast England. B.1.1.7 viruses possess a striking constellation of nucleotide changes with an unusual number of missense mutations in the spike protein of potential biological relevance (3) (Fig. 1). For this reason, lineage B.1.1.7, was designated SARS-CoV-2 variant of concern (VOC) 202012/01 by Public Health England (PHE) since the mutations found might impact transmission, immune response, and disease severity. Mutation N501Y, located within the receptor-binding domain (RBD), can increase binding affinity to human and murine angiotensin-converting enzyme 2 (ACE-2) receptor and cell infectivity in mice (4–6). Substitution P681H is immediately adjacent to the furin cleavage site, important for viral pathogenesis (7, 8), whereas deletion of amino acids 69 and 70 has been identified in multiple lineages associated with different RBD mutations and has been related to the evasion to human immune response (9). The deletion of nucleotides 21765 to 21770 coding for amino acids 69 and 70 has been associated with S-gene target failure (SGTF) in diagnostic PCR testing of samples showing positive results for other gene targets (1) and is used to estimate the proportion of COVID-19 confirmed cases likely associated with infection by variant B.1.1.7. Epidemiological evidence and phylogenetic analysis of viral sequences suggest that viruses of the B.1.1.7 lineage may have increased transmissibility compared to ancestral isolates (2). Whole-genome sequencing and SGTF data have shown a rapid increase in the prevalence of B.1.1.7 variant through time with the proportion of sequences from clinical samples associated with B.1.1.7 in England, reaching around 94% during the last week of January 2021 (1, 2, 10). Additional analyses indicate exponential growth of lineage B.1.1.7 and an increase of between 0.4 and 0.7 in the reproduction number with respect to previous lineages (2).

Other VOCs have been identified recently, notably lineages B.1.351 and P.1, known to be circulating in South Africa since early October 2020 (11) and in Brazil since early December 2020 (12), respectively, and also containing complex mutation constellations, including an N501Y amino acid change (Fig. 1). B.1.351 and P.1 variants have two more spike mutations of biological significance: E484K and K417N/T, both located in the RBD and both important for ACE-2 binding and antibody recognition. Mutations at spike amino acid 484 have been shown to reduce binding to convalescent-phase serum antibodies, with neutralization by some sera reduced >10-fold (13). Amino acid substitutions at residue 417, K417N in B.1.351 and K417T in P.1, appear to improve evasion from antibodies in combination with N501Y and E484K (14). More recently, mutation E484K has been identified in a small number of B.1.1.7 viruses in England (15), raising increased concerns about this variant. The effects of mutations found in the VOCs detailed above suggest that VOCs could be a threat to the protective efficacy of current vaccines by reducing vaccine-induced immunity against them. Recent research has shown that while B.1.1.7 is modestly resistant to sera from vaccinees, B.1.351 is markedly more resistant to neutralization by such sera, and some COVID-19 vaccines appear to show reduced efficacy against this variant (16–20). Although further research is required to fully understand the effect of spike mutations on vaccine efficacy, updating vaccine strains might be required in the future in a similar manner to what is common practice for flu vaccines.

The appearance and rapid growth of VOCs shows the need for enhanced genomic and epidemiological surveillance worldwide to ensure that any new changes in the spike protein that might arise are rapidly detected and their biological effects investigated. Environmental surveillance (ES) has proven to be a sensitive method for the detection and monitoring of SARS-CoV-2 circulation, as described in multiple reports from many countries (reviewed in reference 21). Since ES can produce a real-time snapshot of virus transmission at a given time point, including that from asymptomatic infections, it might provide precise information on the prevalence of VOCs in the population. We were able to detect SARS-CoV-2 viral RNA in sewage samples collected in London during the first wave of the pandemic with variation in viral RNA levels comparing to that of numbers of confirmed COVID-19 cases (22). In addition, using next-generation sequencing (NGS) analysis of PCR amplicons generated from sewage concentrates, we were able to track changes in variant predominance during the first stages of the pandemic detecting variants that were particularly prevalent in the UK and the rapid expansion of D614G variant reaching nearly complete dominance in May 2020 (22). This variant contains a mutation from A to G at nucleotide 23403, resulting in amino acid change from aspartic acid to glycine at residue 614 of the spike protein, which appeared to increase viral infectivity and transmissibility. Variant D614G was first described in late February 2020 in Italy, rapidly expanding and becoming the dominant SARS-CoV-2 variant globally a few months later (23). Similar work by other groups has described the detection of cocirculating SARS-CoV-2 variants in sewage samples from different locations, including a recent report describing the identification of B.1.1.7 lineage in sewage samples from Switzerland (24–28).

In order to further evaluate the value of ES for SARS-CoV-2 detection, we analyzed sewage samples collected in London between 14 January 2020 and 26 January 2021 for the presence of SARS-CoV-2 by using a semiquantitative nested reverse transcription-PCR (nPCR) system targeting two different genomic regions since we have found this system to be more sensitive and consistent than quantitative RT-PCR assays (22). More importantly, we have designed new nPCR reactions to generate DNA amplicons and analyze them by NGS in order to specifically detect and quantify the presence of key mutations that discriminate lineages B.1.1.7, B1.351, and P.1 from ancestral isolates and between themselves.

## RESULTS

### nPCR amplifications to estimate SARS-CoV-2 RNA levels in sewage samples.

**FIG 2:**
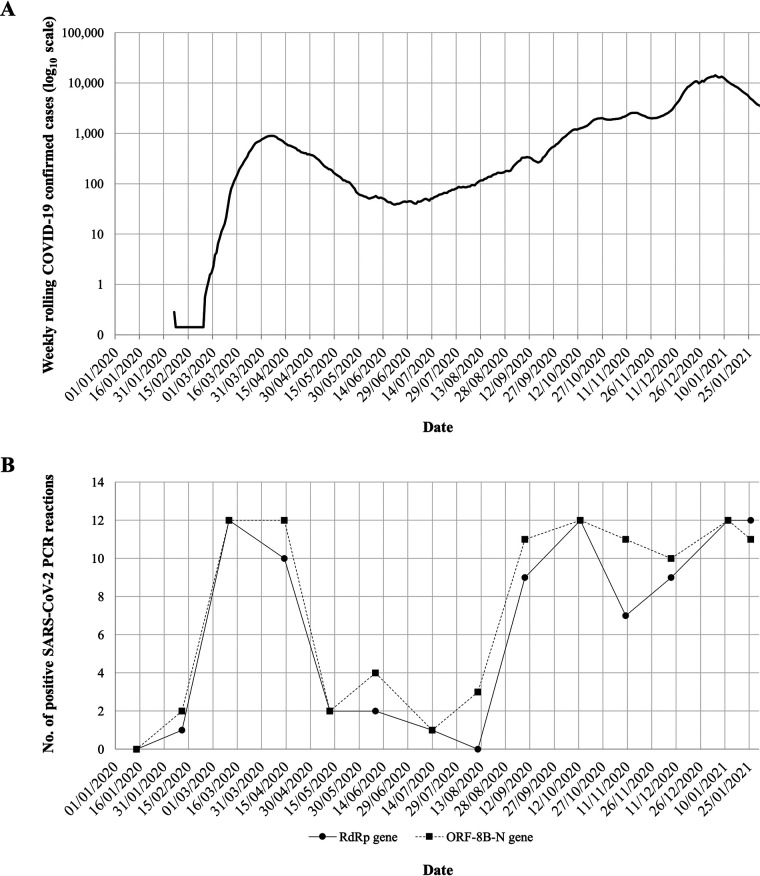
(A) Number of daily confirmed COVID-19 cases in London. Values are represented as 7-day rolling averages. Nationwide lockdown periods are indicated as horizontal lines. The source for the COVID-19 case data is available online (https://coronavirus.data.gov.uk/). (B) Detection of SARS-CoV-2 RNA in sewage samples collected in London between 14 January 2020 and 26 January 2021. The numbers of RNA replicates, out of a total of 12, producing positive results in nPCR A (circles) and nPCR B (squares) reactions targeting RdRP and ORF8b genomic regions, respectively, are represented for each sample date.

Twelve RNA replicates from each sewage concentrate were used to generate nPCR products with primer combinations A and B (see Table S1 in the supplemental material) targeting RNA-dependent RNA polymerase (RdRP) and ORF8b gene regions, respectively. Fourteen sewage samples collected between 14 January 2020 and 26 January 2021 were analyzed. The proportion of positive PCR results for each sample was used as a semiquantitative comparison of RNA levels present in sewage samples. The results are shown in Fig. 2 in the context of epidemiological data. As we have shown before, a sample from 14 January 2020 was negative, and only low levels of viral RNA were detected in the sample from 11 February 2020 (22), 3 days before the first COVID-19 cases were confirmed in the area. The number of RNA replicates producing positive SARS-CoV-2 PCR results increased in March and April, likely reflecting an increase in viral concentration in sewage during the first wave of the pandemic and sharply decreased in May, in agreement with the reduction in COVID-19 cases as a result of nationwide lockdown measures introduced from 23 March. After a period of about 4 months with low-level virus transmission, COVID-19 confirmed cases increased again from early September and so did the number of RNA replicates from sewage samples showing positive SARS-CoV-2 PCR results.

10.1128/mSystems.00353-21.2 TABLE S1 Primers used for nPCR reactions. The location in the SARS-CoV-2 genome sequence of primers used for the nPCR reactions is shown. Download Table S1, PDF file, 0.05 MB. © Crown copyright 2021. 2021 Crown https://creativecommons.org/licenses/by/4.0/ This content is distributed under the terms of the Creative Commons Attribution 4.0 International license.

### NGS analysis of nPCR products for the detection of SARS-CoV-2 B.1.1.7, B1.351, and P.1 lineages.

**FIG 3:**
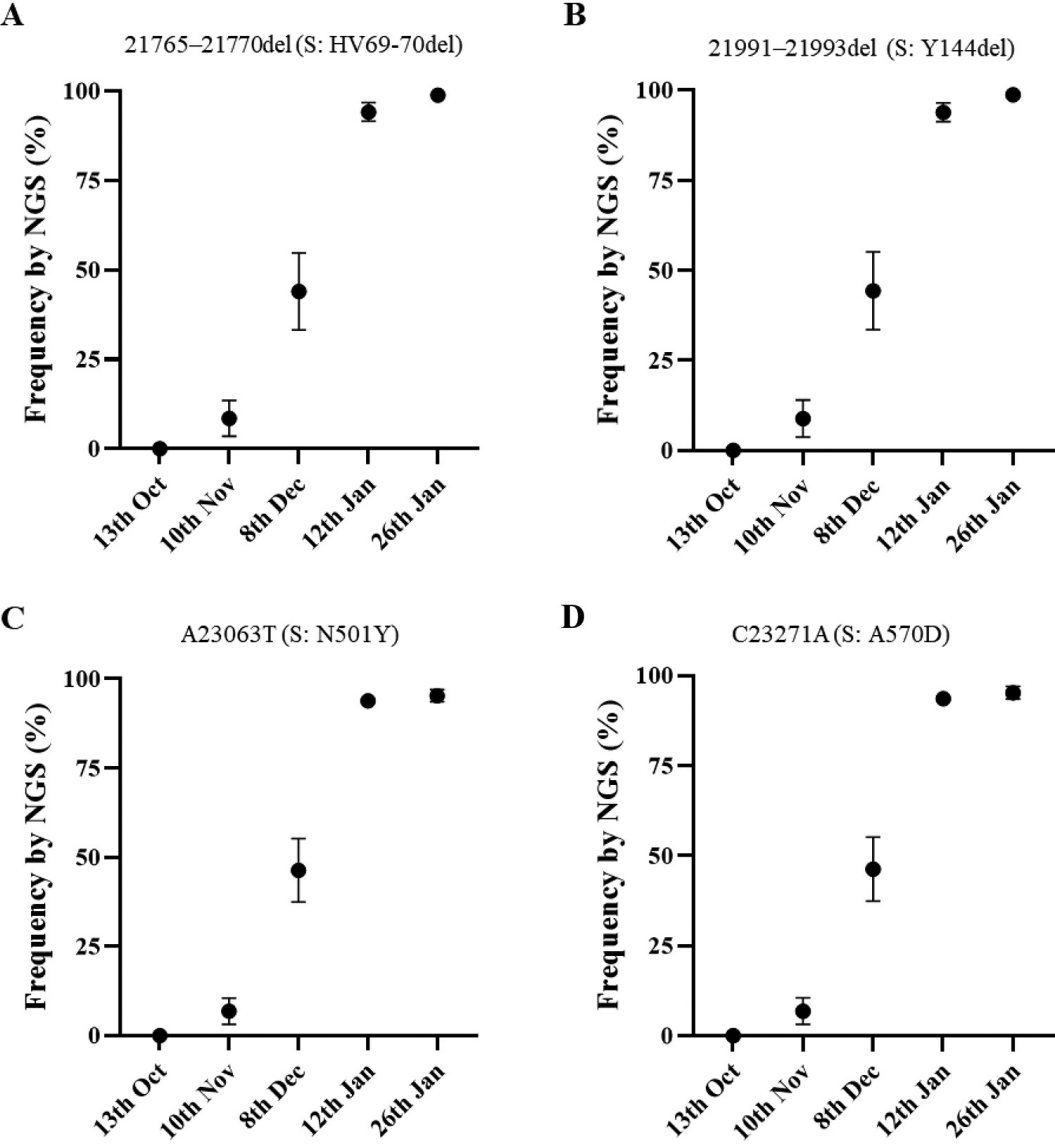
Mean frequency values of nucleotide mutations in the spike gene distinctive of SARS-CoV-2 B.1.1.7 lineage identified in nPCR C and nPCR D products synthesized from sewage concentrates and measured by NGS analysis. (A) Deletion of nucleotides 21765 to 21770 (deletion of amino acids HV69-70); (B) deletion of nucleotides 21991 to 21993 (deletion of amino acid Y144); (C) mutation A23063T (amino acid change N501Y); (D) mutation C23271A (amino acid change A570D). Error bars indicate standard errors of the mean. Mean numeric values are shown in Table 2. Statistical values associated with these data are shown in Table S2.

Twelve additional RNA replicates from each sewage concentrate were used to generate nPCR products with primer combinations C and D (see Table S1), mapping in the spike protein gene. All amplicons from positive nPCR reactions were analyzed by NGS with an aim to detect and quantify nucleotide sequence variations specifically found in B.1.1.7, B1.351, and P.1 lineages (Table 1). Nucleotide sequences characteristic of lineage B.1.1.7 were detected in sewage samples from 10 November 2020 onward. Mean sequence frequency values at key nucleotide positions, calculated from all sequenced replicates for each monthly sewage sample, are shown in Fig. 3. The results show that mutations discriminating the B.1.1.7 lineage from ancestral isolates, which include relevant mutations coding for amino acid changes and amino acid deletions (Table 1), clearly increased in frequency between November 2020 and January 2021 from 6.8 to 8.9% on 10 November to 44.0 to 46.4% on 8 December 2020, 93.6 to 94.1% on 12 January 2021, and 95.2 to 98.8% on 26 January 2021. It is worth noting that all four mutations analyzed showed very similar frequencies between them at any given time, reinforcing the conclusion that they all correspond to an increment of B.1.1.7 lineage through time. An example of NGS results showing an increasing proportion of a nucleotide 21765-21770 deletion (deletion of amino acids HV69-70) in viral RNAs from sequential sewage samples is shown in Fig. S1. It is important to emphasize that when analyzing surveillance data in real time, clinical surveillance has a potential limitation with respect to environmental surveillance since only a fraction of clinical samples is subjected to whole-genome sequence analysis. If 5% or fewer positive clinical samples are sequenced and the prevalence of B.1.1.7 or any other variant of interest in the population is 5%, at least 400 samples would need to be sequenced to find one such virus by chance. The time lapse between infection and symptoms developing, as well as samples taken and being tested, would add further delays compared to the expected immediacy of results from sewage surveillance.

**TABLE 1:** Nucleotide and amino acid differences between VOCs and ancestral isolates^*a*^

Reaction	Genome position		nt				aa			
	nt	aa	WT	B.1.1.7	B.1.351	P.1	WT	B.1.1.7	B.1.351	P.1
nPCR C	21765–21770	69–70	ACATGT	*del*	ACATGT	ACATGT	HV	*del*	HV	HV
	21801	80	A	A	*C*	A	D	D	*A*	D
	21974	138	G	G	G	*T*	D	D	D	*Y*
	21991–21993	144	TTA	*del*	TTA	TTA	Y	*del*	Y	Y
nPCR D	23012	484	G	G	*A*	*A*	E	E	*K*	*K*
	23063	501	A	*T*	*T*	*T*	N	*Y*	*Y*	*Y*
	23271	570	C	*A*	C	C	A	*D*	A	A

a WT, Wuhan-Hu-1 strain (GenBank accession number MN908947). This strain was first detected in the UK (B.1.1.7), South Africa (B.1.351), or Brazil (P.1). Single-letter amino acid codes are used. Changes from the WT sequence are italicized and underlined. Primers for nPCR reactions are shown in Table S1 in the supplemental material. Abbreviations: nt, nucleotides; aa, amino acids.

10.1128/mSystems.00353-21.1 FIG S1 Partial view of NGS reads pile-up showing different proportions of nucleotides 21765 to 21770 deletion (deletion of amino acids HV69-70) in amplicons from sewage concentrates from different dates. Filtered NGS reads were aligned to SARS-CoV-2 reference sequence (in color). Download FIG S1, PDF file, 0.7 MB. © Crown copyright 2021. 2021 Crown https://creativecommons.org/licenses/by/4.0/ This content is distributed under the terms of the Creative Commons Attribution 4.0 International license.

10.1128/mSystems.00353-21.3 TABLE S2 Statistical values of B.1.1.7 frequency data analysis from sewage samples. Download Table S2, PDF file, 0.1 MB. © Crown copyright 2021. 2021 Crown https://creativecommons.org/licenses/by/4.0/ This content is distributed under the terms of the Creative Commons Attribution 4.0 International license.

**FIG 4:**
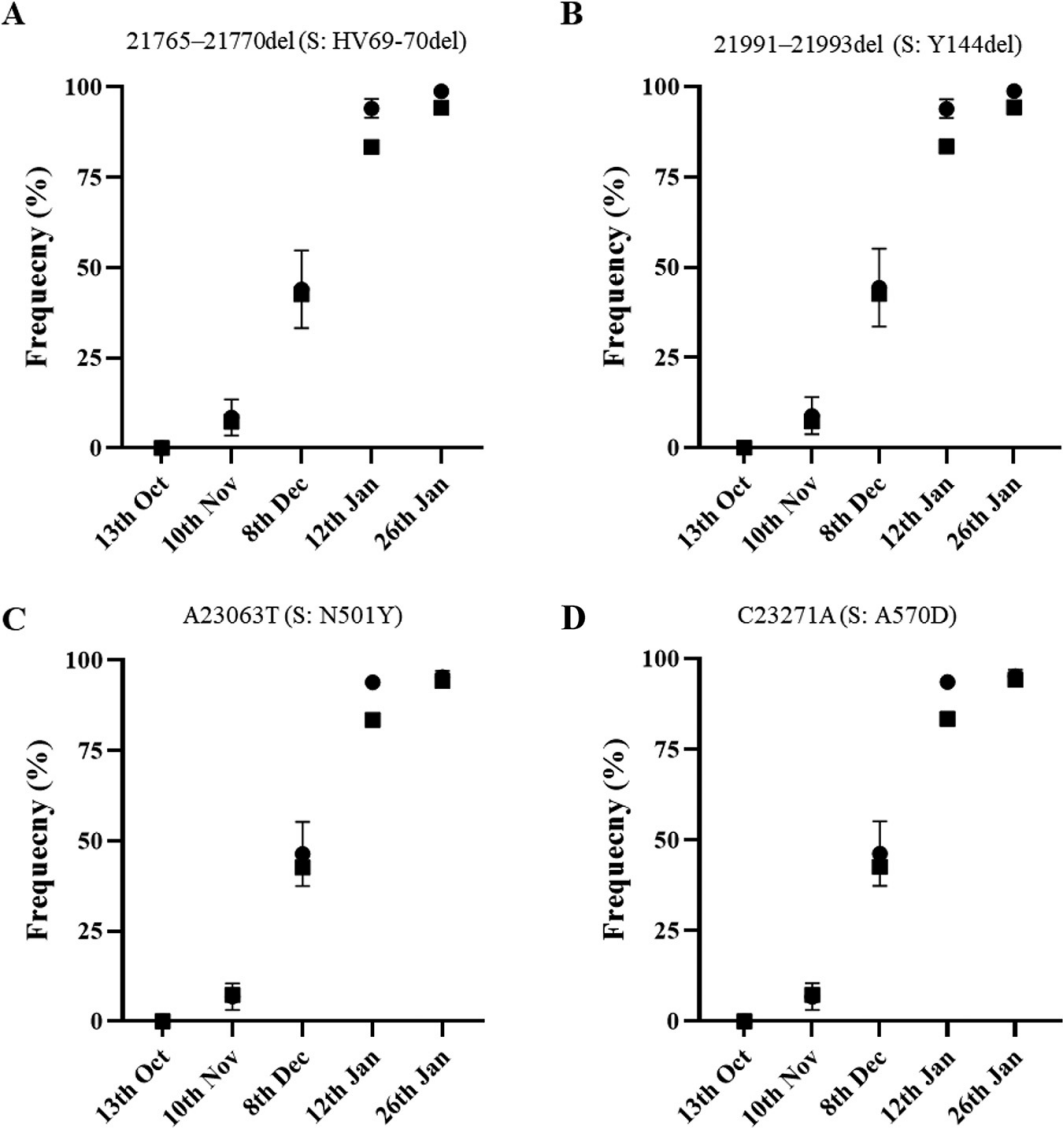
Frequency rates of B.1.1.7 whole-genome sequences among clinical samples from England collected during the week immediately after sewage sample collection are shown as black squares superimposed to mean frequency values of B.1.1.7 mutations found in sewage samples measured by NGS (black circles with error bars indicating standard error of the mean, as shown in Fig. 3). Values in this figure represent data in Table 2. Statistical values associated with these data are shown in Table 2 and Table S3. Panels A to D are as defined for Fig. 3.

Estimates of B.1.1.7 prevalence based on NGS data were in very good agreement with those from whole-genome sequencing and SGTF data from clinical samples from London and England (1, 2); according to the GISAID sequence database (10), the frequency rates of B.1.1.7 sequences from England during the week before and the week after sewage sample collection were 3.4 and 7.3% for 10 November, 25.2 and 42.7% for 8 December, 81.1 and 83.4% for 12 January, and 89.6 and 94.3% for 26 January, which is very much in line with what was observed in sewage (Table 2). No significant differences were found between B.1.1.7 frequency estimates obtained from sewage using data from any of the four mutations analyzed for any of the five sampling dates (*t* test, *P* > 0.05) (see Table S3). A good correlation between B.1.1.7 frequency estimates from mutation analysis of viral RNAs from sewage and clinical data was observed throughout the period of study, with B.1.1.7 frequency increasing from <0.1% in October to >95% in late January and *r*^2^ values ranging between 0.968 and 0.997 (Table 2). There was better agreement between sewage and clinical data when using data from clinical samples corresponding to the week after sewage sample collection than to the week before, with sewage and clinical data superimposing almost perfectly when represented in the same graph (Fig. 4) and small differences between B.1.1.7 frequency estimates from sewage and clinical data representing only between 1.04 and 17.30% of the actual sewage frequency values versus between 5.92 and 61.46% when using clinical data from the week before (Table 2). In any case, this interpretation has to be taken with caution because sewage data corresponding to London, with a catchment area of approximately 4.0 × 10^6^ people, were compared to sequencing data from the whole of England, since no sequence data specific for London are publicly available. Because different regions showed different timelines of B.1.1.7 introduction and growth, the comparison presented above might not be totally accurate. However, results from recent reports show that whole-genome sequencing and SGTF data from clinical samples from London resemble those for the whole of England very closely (1, 2).

**TABLE 2:** Frequency of SARS-CoV-2 B.1.1.7 lineage in sewage (ES) and clinical samples^*a*^

Sample		Date (day.mo.yr)	% B.1.1.7 mutations [SEM] (dif)^*b*^			
			21765–21770 deletion	21991–21993 deletion	A23063T	C23271A
Oct	ES	13.10.20	0.00 [0.00]	0.00 [0.00]	0.00 [0.00]	0.00 [0.00]
	Clinical.1	06–12.10.20	0.08 (0.08)	0.08 (0.08)	0.08 (0.08)	0.08 (0.08)
	Clinical.2	13–19.10.20	0.06 (0.06)	0.06 (0.06)	0.06 (0.06)	0.06 (0.06)
Nov	ES	10.11.20	8.53 [5.01]	8.88 [5.10]	6.88 [3.70]	6.84 [3.69]
	Clinical.1	03–09.11.20	3.42 (5.11)	3.42 (5.46)	3.42 (3.46)	3.42 (3.42)
	Clinical.2	10–16.11.20	7.34 (1.19)	7.34 (1.54)	7.34 (0.47)	7.34 (0.50)
Dec	ES	8.12.20	43.99 [10.77]	44.34 [10.81]	46.36 [8.86]	46.28 [8.85]
	Clinical.1	01–07.12.20	25.19 (18.80)	25.19 (19.15)	25.19 (21.17)	25.19 (21.09)
	Clinical.2	08–14.12.20	42.67 (1.32)	42.67 (1.67)	42.67 (3.69)	42.67 (3.61)
Jan.1	ES	12.01.21	94.13 [2.59]	93.9 [2.58]	93.86 [0.89]	93.6 [0.89]
	Clinical.1	05–11.01.21	81.08 (13.05)	81.08 (12.82)	81.08 (12.78)	81.08 (12.52)
	Clinical.2	12–18.01.21	83.44 (10.69)	83.44 (10.46)	83.44 (10.42)	83.44 (10.16)
Jan.2	ES	26.01.21	98.83 [0.45]	98.78 [0.44]	95.3 [1.71]	95.25 [1.73]
	Clinical.1	19–25.01.21	89.61 (9.22)	89.61 (9.17)	89.61 (5.69)	89.61 (5.64)
	Clinical.2	26.01.21 to 01.02.21	94.26 (4.57)	94.26 (4.52)	94.26 (1.04)	94.26 (0.99)
*r*^2^^*c*^	ES vs Clinical.1		0.980	0.979	0.968	0.968
	ES vs Clinical.2		0.996	0.997	0.994	0.994

a Data were obtained from the GISAID sequence database (10), consulted on 13 February 2021.

b Mean frequency values of B.1.1.7 mutations with associated standard errors of the mean (indicated in brackets) are presented. “dif” indicates the difference between B.1.1.7. mutation frequencies determined in sewage versus the clinical data in absolute number. dif values are given in parentheses.

c *r*^2^ values were obtained from linear regression analysis.

10.1128/mSystems.00353-21.4 TABLE S3 Statistical comparison between B.1.1.7 mutation frequencies determined in viral RNAs purified from sewage samples. *P* value estimates from unpaired *t* test are presented. The statistical significance was determined using the Holm-Sidak method, with α = 0.05. All paired comparisons were found to be nonsignificant. Download Table S3, PDF file, 0.10 MB. © Crown copyright 2021. 2021 Crown https://creativecommons.org/licenses/by/4.0/ This content is distributed under the terms of the Creative Commons Attribution 4.0 International license.

**FIG 5:**
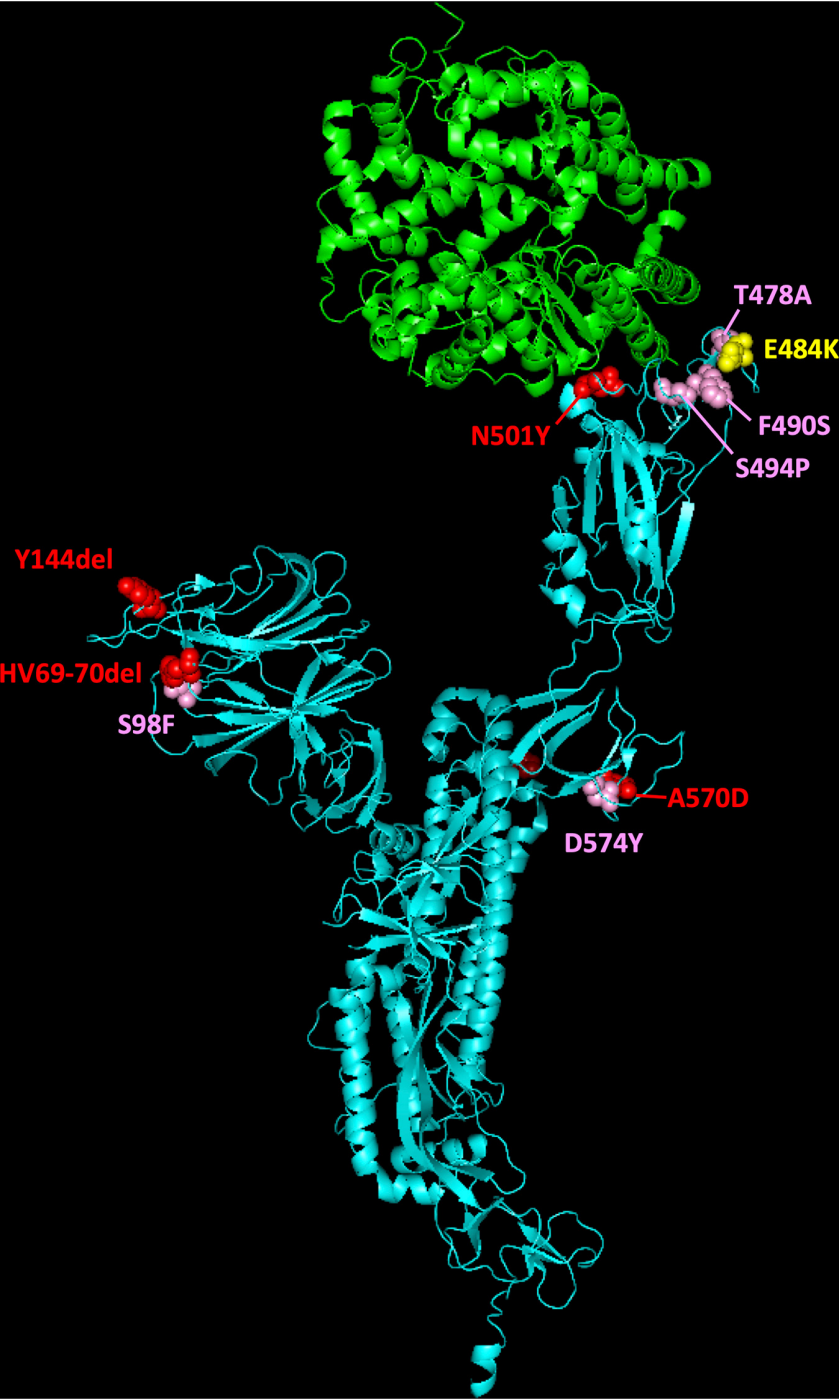
Molecular cartoon diagram of the 3D structure of SARS-CoV-2 spike protein monomer in the open form (cyan) in complex with receptor ACE-2 (green). The image was generated using PyMOL Molecular Graphics System version 1.7.0.3 software (Schrödinger, LLC) using cryo-EM data (Protein Data Bank accession number 7DF4 [36]). The location of selected amino acid substitutions found in SARS-CoV-2 B.1.1.7 variant are shown in red; mutations found at low frequency in viral RNAs from sewage are indicated in pink, and the position of mutation E484K present in B.1.351 and P.1 VOCs is shown in yellow.

In contrast, none of the nucleotide substitutions unique to B.1.351 and P.1 lineages (Table 1) were detected in any of the replicate nPCR products from any of the sewage samples. Additional nucleotide sequence variations were found at different sites in amplicons from sewage samples (Table 3), but none followed the same pattern as that observed with B.1.1.7 mutations, which were repeatedly detected in replicate viral RNA sequences and consistently found in sequential samples in increasing proportions. Interestingly, additional sequence variations most frequently found in viral RNA replicates from the same or different sampling dates mapped at locations adjacent to mutations characteristic of variant B.1.1.7 in the spike protein three-dimensional (3D) structure (Fig. 5): mutation S98F is located next to amino acid 69, which is deleted in variant B.1.1.7; substitution D574Y maps next to amino acid 570, which changes from alanine (A) to aspartic acid (D) in B.1.1.7 viruses; and mutations T478A, F490S, and S494P locate in the RBD, near amino acid 501, which changes from asparagine (N) to tyrosine (Y) in B.1.1.7. Most of these mutations have been found in clinical isolates from England, some of them in relatively large numbers, such as S98F and S494P (Table 3).

**TABLE 3:** Additional missense nucleotide variations identified in viral RNAs from sewage samples

Sample	nt change	aa change	SNP (%)^*a*^	Clinical sequences^*b*^		
				Total UK (no.)	Last 28 days England (no.)	Date of first detection
13-Oct-20	A22994G	T478A	7.8, 7.6			
	A23201G	T547A	7.8	1	0	23-10-20
	G23282T	D574Y	99.4, 44.4	400	5	30-03-20
10-Nov-20	T21710C	S50P	8.4			
	T21765C	I68T	12.1	19	0	10-04-20
	C21846T	T95I	7.8	456	141	22-03-20
	C21855T	S98F	70.1, 14.5	1,672	281	22-03-20
	C21867A	R102K	6.5			
	C22995G	T478R	18.3	22	0	19-10-20
	T23105C	F515L	6.5	1	0	01-01-21
	G23282T	D574Y	33.1, 14.2	400	5	30-03-20
8-Dec-20	C21727T	S56F	10.0			
	A21894G	D111G	11.8			
	T21990C	V143A	5.1			
	T23031C	F490S	7.3	100	50	05-04-20
	T23075C	Y505H	8.2	9	4	02-11-20
12-Jan-21	A21683G	K41E	18.2	2	0	27-11-21
	T21723C	L54S	5.1	1	0	29-01-21
	G21761T	A67S	99.6	149	28	19-04-20
	A21779G	T73A	16.3	4	2	16-10-20
	C21855T	S98F	6.0, 48.0	1,672	281	22-03-20
	T21861C	I100T	31.9	15	0	27-03-20
26-Jan-21	T21831C	V90A	13.2			
	C21855T	S98F	6.3, 5.9, 16.4	1,672	281	22-03-20
	A21975G	D138G	13.2	25	18	13-10-20
	T23031C	F490S	7.0	100	50	05-04-20
	T23042C	S494P	5.1, 5.7, 6.8	740	162	20-03-20
	C23202A	T547I	7.8	87	20	07-04-20

a The percentages of mutations found in 12 individual replicate amplicon sequences per sewage sample are presented. Results are shown for each replicate in which the mutation was detected.

b Numbers and dates for clinical isolates showing the mutation are presnted. The results were obtained using the COVID-19 Genomics (COG-UK) Consortium/Mutation Explorer from sequencing data generated on 28 February 2021 (http://sars2.cvr.gla.ac.uk/cog-uk/). Variations found in more than one replicate from the same or different collection dates are underlined.

### Sanger sequence analysis of nPCR products.

Selected PCR products from nPCR reactions A and B were analyzed by Sanger sequencing to confirm the SARS-CoV-2 genome sequence. nPCR product B contains codon GAT at nucleotide position 28280, which changes to CTA in B.1.1.7 lineage viruses, resulting in the amino acid substitution aspartic acid to leucine at residue 3 of the nucleocapsid (N) protein. Hence, nPCR product B can also be used to identify and quantify B.1.1.7 sequences in sewage samples. The Sanger sequence analysis revealed that B.1.1.7 codon CTA was not present in replicate viral sequences from sewage concentrates from September (0/6) and October 2020 (0/4) but was detected in replicate samples from November 2020 (1/4), December 2020 (2/3), and January 2021 (2/2) in agreement with the results of NGS analysis of nPCR products C and D described above.

## DISCUSSION

We were able to detect SARS-CoV-2 RNA in sewage samples from London throughout the COVID-19 pandemic, with changes in viral RNA levels shown to be in good agreement with those of confirmed COVID-19 cases reflecting national restrictions (Fig. 2). The second lockdown period lasting from 5 November to 2 December appear to only have had a limited effect in reducing virus transmission which, together with the emergence of the apparently highly transmissible B.1.1.7 variant, may explain the high levels of viral RNA found in sewage and the high number of SARS-CoV-2 infections reported in England in December 2020 and January 2021. In England 1 in 50 people (1 in 30 in London) were estimated to have had SARS-CoV-2 infection in the week ending 2 January according to an infection survey by the Office for National Statistics (29). The third nationwide lockdown initiated on 4 January 2021 is having a clear effect at reducing virus transmission, as judged by the observed drop in COVID-19 confirmed cases, but as of 26 January 2021, SARS-CoV-2 RNA levels remained high in sewage (Fig. 2), reflecting the extent of virus transmission occurring during the second phase of the second wave of the COVID-19 epidemic. Although detailed quantification of SARS-CoV-2 RNA in sewage and its correlation with COVID-19 infection prevalence was outside the scope of the present study and would require more frequent and widespread sampling, our results suggest that high viral RNA concentration levels could be detected preceding peaks in COVID-19 confirmed cases, showing the potential predictive value of ES, as has been shown in other studies (21).

NGS analysis of DNA amplicons directly synthesized from SARS-CoV-2 RNA present in sewage concentrates from London allowed us to readily identify viruses belonging to B.1.1.7 lineage before widespread transmission of this variant was obvious. We were able to detect B.1.1.7 variant at a frequency of 6.8 to 8.9% in a sample collected on 10 November 2020, a few weeks before it was first noticed through clinical surveillance. The B.1.1.7 variant first became apparent in late November 2020 when PHE was investigating why infection rates in Kent remained high despite national restrictions. A concerning cluster of virus isolates showing an unusual constellation of mutations was then found that could explain the persistent high infection rates (3, 15). Retrospective analysis showed that the two earliest genomes belonging to B.1.1.7 lineage were collected on 20 September and 21 September 2020 in Kent and London, respectively. B.1.1.7 is believed to have circulated at very low levels in the population until mid-November; it then rapidly spread across the country. The frequency rates of B.1.1.7 sequences observed in sewage between November 2020 and January 2021 were very similar to those of COVID-19 cases associated with infection by B.1.1.7 viruses at around the time of sewage sample collection (Table 2). B.1.1.7 was not detected in the sewage sample from 13 October 2020, consistent with the fact that the frequency of B.1.1.7 viruses identified in clinical samples collected in the weeks before and after 13 October 2020 was <0.08% in the whole of England. The proportion of B.1.1.7 sequences found in sewage increased rapidly from early November, reaching >90% 8 weeks after the first detection, in agreement with increasing infections associated with B.1.1.7. No evidence of the presence of variants B.1.351 and P.1 was found in any of the sewage samples, which was expected since only a small number of B.1.351 isolates had been found in England at that time, not necessarily in London, and no P.1 viruses had been detected in the UK yet. The G23012A nucleotide substitution present in both B.1.351 and P.1 lineages and responsible for spike amino acid change E484K, thought to play a critical role at changing virus antigenicity and reducing human immune response to the virus and vaccine efficacy (13, 16–20), was not detected in viral genomes from sewage. This mutation has recently been found in a small number of B.1.1.7 isolates in England but mostly in locations away from London (15). We expect that the methods shown here can help to rapidly identify such mutants, as well as B.1.351 and P.1 lineages, in the same way that we have identified B.1.1.7 viruses (Table 1). We did find additional mutations in low frequency at amino acid sites of possible biological relevance located close to mutations characteristic of variant B.1.1.7 in the spike protein 3D structure; most of these are known to be present in clinical isolates from England. Mutations T478A, F490S, and S494P, in particular, are located very close to amino acid 484, known to have an effect on virus antigenicity, as discussed above. Similarly, mutations at these three sites have been shown to be associated with weaker neutralization to the virus by sera from convalescent people who have been infected with SARS-CoV-2 and/or monoclonal antibodies that could be used to patients with COVID-19 (30–34). It is important to monitor the appearance and to quantify the prevalence of such additional mutations since they might represent a challenge for vaccine efficacy. As shown in this study, ES appears to be a suitable system for such purpose. An early assessment of the biological effect of mutations of concern found to be prevalent in successive samples would be critical.

These methods can also be used to sequence SARS-CoV-2 in clinical samples using traditional Sanger sequencing for the rapid identification of VOCs in settings where whole-genome sequencing capabilities are limited. New PCRs can be quickly designed to track down new VOCs that might arise anywhere and detect importations. Furthermore, systematic sequencing of the spike gene of viral RNAs present in sewage samples collected regularly would help in proactively identifying potential new VOCs based on mutations and trends encountered. Eventually, if vaccine strains need to be regularly updated, as is current practice with flu vaccines, ES could be a useful resource to help vaccine strain selection based on prevalent SARS-CoV-2 variants circulating around the world.

As shown here, ES has a potential advantage for the early detection of VOCs versus clinical surveillance because it provides an immediate snapshot of virus transmission events representing thousands/millions of people, including asymptomatic infections. The methods shown here can provide sequencing results within 1 week of sewage samples arriving in the laboratory. According to the GISAID sequence database (10), only 33 B.1.1.7 sequences had been identified on 16 November 2020 in England, 1 week after the collection date of our first positive sewage sample. Even in the context of the phenomenal SARS-CoV-2 sequencing program established in England, clinical surveillance requires some time to build up enough data representative of current transmission patterns that is comparable to that provided by ES. In addition, since most clinical testing is based on samples from symptomatic infections, a delay of several days is expected between initial infection and viral sequence data availability due to the time required for symptoms to develop, samples to be collected, and PCR/sequencing analyses to be completed. Clinical surveillance is highly dependable on rapid and widespread PCR testing, as well as an extensive whole-genome sequencing program. This is currently only happening in a few countries so, in most locations, ES could be a major asset for monitoring SARS-CoV-2 virus circulation and identifying VOCs. After preparation of this manuscript, we recently detected a clear decline in the proportion of B.1.1.7 lineage in a sewage sample from April 2021 for the first time since it became dominant in late January 2020 and a concurrent increase in the presence of non-B.1.1.7 VOCs such as B.1.351 and B.1.525 (further research is in progress). On the other hand, identification of locally circulating VOCs could be delayed using ES at sites that cover very large populations, and therefore community-based ES targeting smaller populations would increase the sensitivity for detection of such VOCs, e.g., current efforts to track down transmission of B.1.1.7 viruses harboring mutation E484K in England could be helped by community-based ES in areas where isolation of such viruses are known to have occurred.

## MATERIALS AND METHODS

### Wastewater sample collection and processing.

One-liter inlet wastewater composite samples were collected during a 24-h period at a sewage plant in London with a catchment area of approximately 4.0 × 10^6^ people. Monthly samples collected every second Tuesday of the month were collected between 14 January 2020 and 12 January 2021, with an additional sample collected on 26 January 2021. Each sample was processed using a filtration-centrifugation method described before (22). Briefly, following removal of solids by centrifugation at 3,000 × *g*, raw sewage was filtered through a 0.45-μm filter (Nalgene 500-ml Rapid-Flow) and concentrated down from 70 ml to 200 to 400 μl using Merck Millipore Centricon Plus-70 centrifugal filter units with a 10-kDa molecular weight cutoff (Merck) according to the manufacturer’s instructions. Samples from January to May 2020 reported previously (22) were reanalyzed in this study.

### nPCR amplifications.

Viral RNA was purified from sewage concentrates using the High-Pure viral RNA kit (Roche). RT-PCR fragments were amplified from purified viral RNAs using an nPCR system described before consisting of a one-step RT-PCR, followed by a second PCR using the first PCR product as the template (22). The genome location and nucleotide sequences of primer sets used for the nPCR reactions are shown in Table S1. Primer sets A and B targeting RdRP and ORF8b gene regions were used for diagnostic nPCRs. Primer sets C and D targeting spike gene regions were used for the detection of B.1.1.7, B.1.351, and P.1 lineages. As shown in Table 1, sequence analysis of nPCR C and D products can discriminate between B.1.1.7, B.1.351, and P.1 lineages and between them and ancestral isolates. Primers were tested using serial dilutions of purified RNA from the National Institute for Biological Standards and Control (NIBSC)’s virus reagent 19/304 containing noninfectious synthetic SARS-CoV-2 RNA packaged within a lentiviral vector (https://www.nibsc.org/products/brm_product_catalogue/detail_page.aspx?catid=19/304). Twelve RNA aliquots from each sewage sample were used for nPCR amplifications for each nPCR reaction. Good laboratory practices were observed in all assays to reduce the possibility of cross-contamination, using different laboratory locations for sample processing, preparation of reaction mixtures, template addition, and postprocessing analysis. RNA extraction and negative template controls were included in every assay. Selected purified DNA amplicon products were sequenced by the Sanger method using an ABI Prism 3130 genetic analyzer (Applied Biosystems).

### NGS analysis of nPCR products.

Sequencing libraries were constructed by A/T adapter ligation using the KAPA HyperPrep kit (Roche, Switzerland) and dual-indexed using IDT TruSeq DNA unique dual indexes (Illumina, USA) with five PCR cycles for library amplification. These libraries were pooled in equimolar concentrations according to manufacturer’s instructions and sequenced with 250-bp paired-end reads on MiSeq v2 (500 cycles) kits (Illumina). Initial demultiplexing was performed on-board by the MiSeq Reporter software. FASTQ sequencing data were adapter and quality trimmed by Cutadapt v2.10 (35) for a minimum Phred score of Q30, minimal read length of 75 bp, and 0 ambiguous nucleotides.

### Generation of SARS-CoV-2 sequence contigs and identification of single nucleotide polymorphisms.

Further processing and analysis of NGS data were performed using Geneious 10.2.3 software as described before (22). Filtered reads were imported into Geneious 10.2.3, paired-end reads were combined and merged, and sequence contigs were built by reference-guided assembly. Reads were mapped to references with a minimum 50-base overlap, minimum overlap identity of 90%, maximum 10% mismatches per read, allowing up to 15% gaps, and index word length of 12. Single nucleotide polymorphisms (SNPs) were identified using Geneious 10.2.3 default settings. The original SARS-CoV-2 Wuhan-Hu-1 strain (GenBank accession number MN908947) was used as a reference. Variants with coverage <500, average quality <30, variant frequency <5%, and strand-bias *P* > 10^−6^ were excluded. The analysis of several amplicon replicates per sample is critical to reduce sampling effects and increase the accuracy of SNP determinations for viral genomes present in a given sewage sample. If we set up a conservative limit of 5% for any SNP found in any replicate amplicon sequence to be accepted as genuinely present in viral RNAs, and given that we sequenced 12 independent RNA replicates per sample and nPCR reaction, the limit of detection for detecting a sequence variant would be approximately 0.42%, which seems reasonably low for the early detection of the spread of SARS-CoV-2 variants of potential concern. If we consider sequencing 5% of all positive clinical samples as a minimum requirement, which is currently only achieved in a few countries, a 0.42% prevalence of a variant would mean finding 21 to 105 viruses among 10,000 to 50,000 new daily positive cases, respectively. However, for clinical surveillance to be representative and have comparable sensitivity to ES, widespread and consistent clinical sampling across the sewage catchment area would be needed.

### SARS-CoV-2 nucleotide sequence analysis and estimates of the prevalence of B.1.1.7 lineage among whole-genome sequences from clinical samples.

SARS-CoV-2 sequences obtained in this study were compared to those available in the Global Initiative on Sharing All Influenza Data (GISAID) SARS-CoV-2 sequence database (10). The COVID-19 Genomics (COG-UK) Consortium/Mutation Explorer with sequencing data generated by 28 February 2021 (http://sars2.cvr.gla.ac.uk/cog-uk/) was also used to examine the presence and proportion of selected viral amino acid variations in clinical samples. Geneious version 10.2.3 software (Biomatters) was used for these analyses. Estimates of the proportion of viruses belonging to B.1.1.7 lineage at different time points in England were obtained using the GISAID SARS-CoV-2 sequence database (10) with results available on 1 March 2021.

### Statistical analysis.

Statistical analysis and graphical representation of variant sequence frequency data were conducted using GraphPad Prism version 8.1.2 software. Statistical significance was determined using the Holm-Sidak method, with alpha = 0.05.

### Data availability.

FASTQ files used in this study are available from the NCBI Short Read Archive under project code PRJNA712959.
