# Supplementary material for: Rapid Increase of SARS-CoV-2 Variant B.1.1.7 Detected in Sewage Samples from England between October 2020 and January 2021
Source: mSystems. 2021 Jun 15;6(3):e00353-21. doi: 10.1128/mSystems.00353-21 (PMC8269227; doi:10.1128/mSystems.00353-21)
Supplement: TABLE S1 [file msystems.00353-21-st001.pdf]

Table S1. Primers used for nested RT-PCR (nPCR) reactions.

Reaction	Primer nt sequence (5'-3')	Target gene [nt.][1]
**nPCR A**	1st reaction	RdRP
forward	GGGTTGGGATTATCCTAAATGTGATAGA	[15,506-15,740]
reverse	GTCCTTTAGTAAGGTCAGTCTCAGTC	
	2nd reaction	
forward	GAGATGCCACAACTGCTTATGC	
reverse	CCACTAGACCTTGAGATGC	
**nPCR B**	1st reaction	ORF8b
forward	AATCATCACAACTGTAGCTGCATTT	[28,120-28,731]
reverse	AACTGTTGCGACTACGTGATGAG	
	2nd reaction	
forward	CACCCATTCAGTACATCGATATCGG	
reverse	GAAGTTGTAGCACGATTGCAGC	
**nPCR C**	1st reaction	Spike
forward	GCCACTAGTCTCTAGTCAGTGTG	[21,683-22.012]
reverse	CTTCAAGGTCCATAAGAAAAGGCTG	
	2nd reaction	
forward	ACACGTGGTGTTTATTACCCTGAC	
reverse	ACTCTGAACTCACTTTCCATCCAAC	
**nPCR D**	1st reaction	Spike
forward	GGCTGCGTTATAGCTTGGAATTC	[22,994-23,293]
reverse	TGAATAGCAACAGGGACTTCTGTG	
	2nd reaction	
forward	CTGAAATCTATCAGGCCGGTAGC	
reverse	TCAAGAATCTCAAGTGTCTGTGGATC	

[1] Extent of SARS-CoV-2 genome PCR fragment sequenced.
