# Supplementary material for: Rapid Increase of SARS-CoV-2 Variant B.1.1.7 Detected in Sewage Samples from England between October 2020 and January 2021
Source: mSystems. 2021 Jun 15;6(3):e00353-21. doi: 10.1128/mSystems.00353-21 (PMC8269227; doi:10.1128/mSystems.00353-21)
Supplement: TABLE S2 [file msystems.00353-21-st002.pdf]

Table S2. Statistical values of B.1.1.7 frequency data analysis from sewage samples.

Mutation	Date	Mean	SD	SEM	Upper 95% CL	Lower 95% CL	N
**HV69-70del**	13/10/2020	0.00	0.00	0.00	0.00	0.00	12
	10/11/2020	8.53	17.36	5.01	19.56	-2.50	12
	08/12/2020	43.99	37.29	10.77	67.69	20.30	12
	12/01/2021	94.13	8.96	2.59	99.82	88.43	12
	26/01/2021	98.83	1.57	0.45	99.83	97.83	12
**Y144del**	13/10/2020	0.00	0.00	0.00	0.00	0.00	12
	10/11/2020	8.88	17.68	5.10	20.11	-2.36	12
	08/12/2020	44.34	37.44	10.81	68.13	20.55	12
	12/01/2021	93.90	8.95	2.58	99.59	88.21	12
	26/01/2021	98.78	1.53	0.44	99.75	97.81	12
**N501Y**	13/10/2020	0.00	0.00	0.00	0.00	0.00	12
	10/11/2020	6.88	12.82	3.70	15.02	-1.27	12
	08/12/2020	46.36	30.68	8.86	65.85	26.86	12
	12/01/2021	93.86	3.09	0.89	95.82	91.89	12
	26/01/2021	95.30	5.91	1.71	99.05	91.55	12
**A570D**	13/10/2020	0.00	0.00	0.00	0.00	0.00	12
	10/11/2020	6.84	12.78	3.69	14.96	-1.28	12
	08/12/2020	46.28	30.66	8.85	65.76	26.79	12
	12/01/2021	93.60	3.09	0.89	95.56	91.64	12
	26/01/2021	95.25	5.99	1.73	99.05	91.45	12

SD: Standard Deviation

SEM: Standard Error of the Mean

CL: Confidence Limit

N: Number of replicates
