# Supplementary material for: Rapid Increase of SARS-CoV-2 Variant B.1.1.7 Detected in Sewage Samples from England between October 2020 and January 2021
Source: mSystems. 2021 Jun 15;6(3):e00353-21. doi: 10.1128/mSystems.00353-21 (PMC8269227; doi:10.1128/mSystems.00353-21)
Supplement: TABLE S3 [file msystems.00353-21-st003.pdf]

Table S3

Statistical comparison between B.1.1.7 mutation frequencies determined in viral RNAs purifed from sewage samples.

		**P-Values**		**Date**
Mutation	**Y144del**	**N501Y**	**A570D**	13-Oct-20
**HV69-70del**	1.00	1.00	1.00	
**Y144del**		1.00	1.00	
**N501Y**			1.00	
	**Y144del**	**N501Y**	**A570D**	10-Nov-20
**HV69-70del**	0.96	0.81	0.81	
**Y144del**		0.78	0.77	
**N501Y**			1.00	
	**Y144del**	**N501Y**	**A570D**	08-Dec-20
**HV69-70del**	0.96	0.74	0.74	
**Y144del**		0.77	0.78	
**N501Y**			0.99	
	**Y144del**	**N501Y**	**A570D**	12-Jan-21
**HV69-70del**	0.98	0.97	0.94	
**Y144del**		0.99	0.97	
**N501Y**			0.97	
	**Y144del**	**N501Y**	**A570D**	26-Jan-21
**HV69-70del**	0.99	0.61	0.61	
**Y144del**		0.62	0.62	
**N501Y**			0.99	
