# Supplementary material for: Rapid Increase of SARS-CoV-2 Variant B.1.1.7 Detected in Sewage Samples from England between October 2020 and January 2021
Source: mSystems. 2021 Jun 15;6(3):e00353-21. doi: 10.1128/mSystems.00353-21 (PMC8269227; doi:10.1128/mSystems.00353-21)
Supplement: FIG S1 [file msystems.00353-21-sf001.pdf]

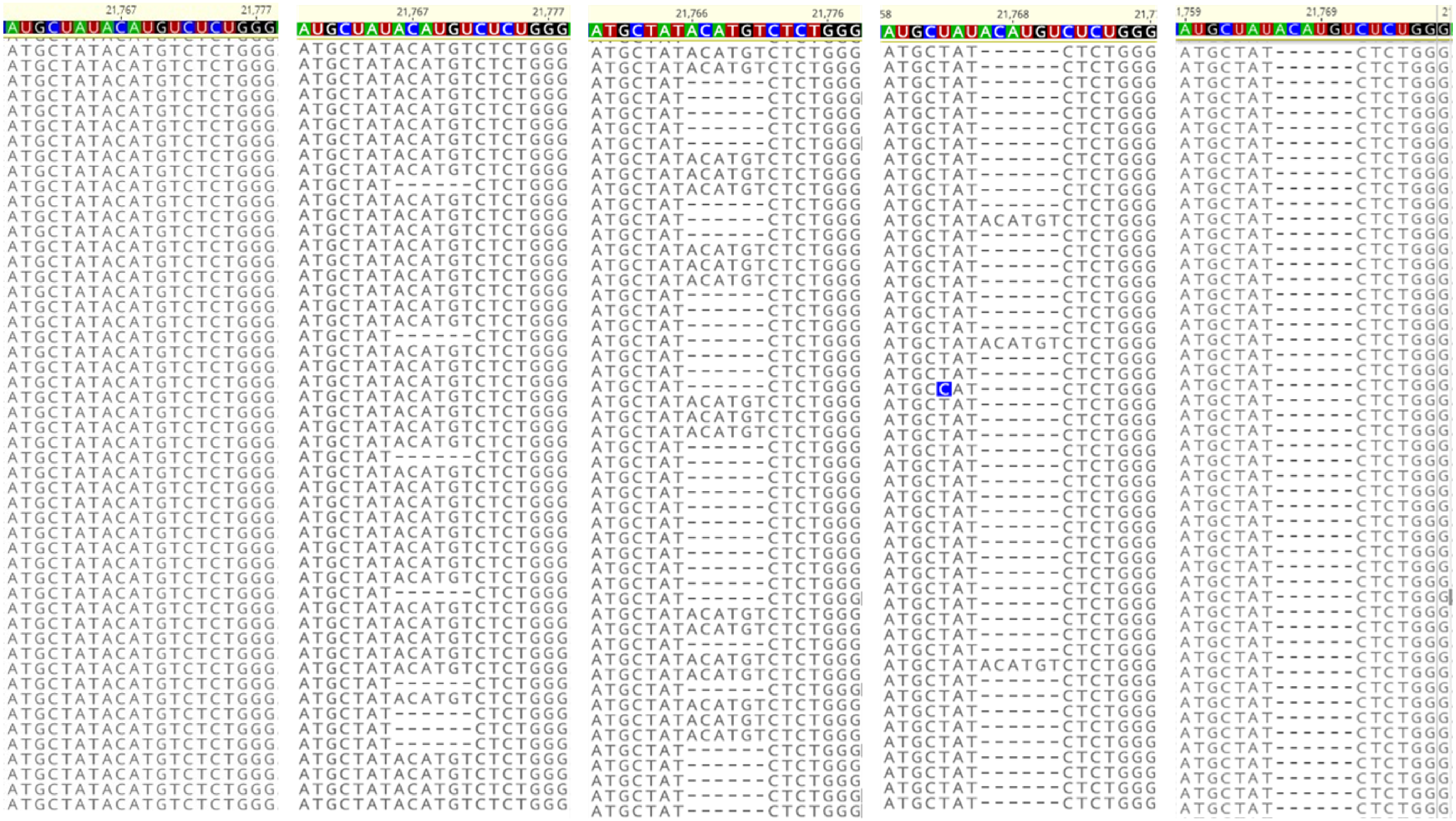

13th October 20 **10th November 20** **8th December 20** **12th January 21** **26th January 21**
